# P04.44. The association between the use of complementary and alternative medicine in children with demographic indicators and vaccinations

**DOI:** 10.1186/1472-6882-12-S1-P314

**Published:** 2012-06-12

**Authors:** N Feiler-Mircus

**Affiliations:** 1 Bar-Ilan University, Herzelia, Israel

## Purpose

The aims of the study were to examine: (1) the relationship between parental income and schooling and using Complementary and Alternative Medicine (CAM) for their children; (2) the relationship between giving the recommended vaccines and usage of CAM for children; and (3) the frequency and characteristics of CAM usage in children from different socio-economic layers.

## Methods

The study was done via a self-reporting anonymous questionnaire by parents of 535 children, in 5 primary care pediatric clinics located in different areas in Israel, representing a population from different socio-economic classes.

## Results

Twenty-seven percent of the respondents reported that they used CAM for at least one child in their family. Parents with higher education and income used CAM more than less educated parents and those with lower incomes. Parents who have tried CAM for themselves used it also for their children. Parents who have not vaccinated or partially vaccinated their children used CAM more than parents who gave their children all of the recommended vaccines. Parents tend to attach importance to the pediatrician notification for using CAM.

## Conclusion

A significant portion of the population use CAM for children. CAM use in children was more frequent among families in a high socio-economic level. Most of the parents who used CAM for themselves did the same for their children, due to their positive experience and their desire to improve the health of their children using other methods outside of conventional medicine. Parents who tend not to vaccinate or partially vaccinate their children were more likely to use CAM, because this treatment is perceived as more “natural". The common use of CAM in children requires raising the awareness and knowledge of pediatricians to this phenomena and improving the communication between parents and pediatricians regarding CAM usage, for the benefit of the children.

